# MiR-35 buffers apoptosis thresholds in the *C. elegans* germline by antagonizing both MAPK and core apoptosis pathways

**DOI:** 10.1038/s41418-019-0325-6

**Published:** 2019-04-05

**Authors:** Anh T. Tran, Eric M. Chapman, Mathieu N. Flamand, Bin Yu, Samuel J. Krempel, Thomas F. Duchaine, Matthew Eroglu, W. Brent Derry

**Affiliations:** 1 Developmental & Stem Cell Biology Program, The Hospital for Sick Children, Toronto, ON M5G 1X8 Canada; 2 Department of Molecular Genetics, University of Toronto, Toronto, ON M5S 1A8 Canada; 3 Department of Biochemistry, McGill University, Montreal, QC H3A 1A3 Canada; 4 Division of Experimental Medicine & Goodman Cancer Research Center, McGill University, Montreal, QC H3A 1A3 Canada; 5 Present Address: Human Health Therapeutics, National Research Council Canada, 100 Sussex Drive, Ottawa, ON K1N 5A2 Canada; 6 Present Address: Department of Biochemistry, Duke University School of Medicine, Durham, NC 27710 USA

**Keywords:** Development, Epigenetics

## Abstract

Apoptosis is a genetically programmed cell death process with profound roles in development and disease. MicroRNAs modulate the expression of many proteins and are often deregulated in human diseases, such as cancer. *C. elegans* germ cells undergo apoptosis in response to genotoxic stress by the combined activities of the core apoptosis and MAPK pathways, but how their signalling thresholds are buffered is an open question. Here we show *mir-35–42* miRNA family play a dual role in antagonizing both NDK-1, a positive regulator of MAPK signalling, and the BH3-only pro-apoptotic protein EGL-1 to regulate the magnitude of DNA damage-induced apoptosis in the *C. elegans* germline. We show that while miR-35 represses EGL-1 by promoting transcript degradation, repression of NDK-1 may be through sequestration of the transcript to inhibit translation. Importantly, dramatic increase in NDK-1 expression was observed in cells about to die. In the absence of miR-35, increased NDK-1 activity enhanced MAPK signalling that lead to significant increases in germ cell death. Our findings demonstrate that NDK-1 acts upstream of (or in parallel to) EGL-1, and that miR-35 targets both *egl-1* and *ndk-1* to fine-tune cell killing in response to genotoxic stress.

## Introduction

Apoptosis is a conserved fundamental biological cell death process critical for development, homoeostasis and stress responses. It has profound effects on diseases, including cancer and neurodegenerative conditions. Despite intense research, how apoptosis is fine-tuned to ensure appropriate level of cell culling remains obscure. The apoptosis programme encompasses three key stages: a signal threshold to elicit death, irreversible commitment to the response, and a time delay that is inversely proportional to signal strength [1]. Understanding how thresholds for induction of apoptosis are established is critical for finding ways in overcoming resistance toward lethal stimuli used to treat diseases such as cancer. Conversely, increasing the apoptotic threshold might help prevent inappropriate cell death in patients with neurodegenerative diseases where excessive apoptosis is pathogenic.

In *C. elegans*, somatic cells undergo apoptosis through lineage-specific transcriptional induction of the BH3-only gene *egl-1*, whereas germ cells can undergo apoptosis in response to stresses such as DNA damage [2], or physiological apoptosis independent of EGL-1 (ref. [3]). The p53-like protein CEP-1 (ref. [4, 5]) induces transcription of *egl-1* in germ cells subject to genotoxic stress [6, 7]. EGL-1 protein binds and inhibits CED-9, BCL-2 like protein, which releases APAF-1 orthologue CED-4 to activate CED-3 caspase [8]. Germ cell apoptosis is licensed by the mitogen-activated protein kinase (MAPK) signalling pathway [3, 9, 10]. Although the magnitude of cell death is proportional to MAPK signalling output [9–12], it remains mysterious how this pathway is buffered to control cell killing.

MicroRNAs (miRNAs) have emerged as important regulators of apoptosis under many conditions, including genotoxic stress [13–15] and function by post-transcriptionally regulating the expression of genes in a plethora of signalling pathways in many organisms [16, 17]. Binding of miRNA to the target 3′-UTR promote translational inhibition and/or transcriptional degradation [18]. miRNA binding can also lead to the translocation of target mRNA into cytoplasmic processing bodies (P-bodies) for translational inhibition. P-bodies may also function as temporary storage centres where mRNAs are held in stasis, spatially removed from the translational machinery [19–21]. One miRNA family of interest is the *mir-35-42*, which is essential for embryonic development, and is highly expressed in oocytes and early embryos [22–24]. The *mir-35* family consists of eight members (*mir-35-42*), and deletion of the entire family leads to a high frequency of embryonic and larval lethality [24]. Deletion of seven of the eight *mir-35* family members (*mir-35-41*) by the *gk262* allele, where only *mir-42* is expressed, causes partially penetrant embryonic lethality [24–26]. The *mir-35* family suppresses somatic cell apoptosis during embryonic development [27], but it is not known if this mode of regulation is relevant to stress-induced apoptosis in the germline.

Here, we uncover a critical role for the *mir-35-42* miRNA family in regulating the magnitude of DNA damage-induced apoptosis in the *C. elegans* germline. Absence of *mir-35-41* results in up to fourfold more cell killing in response to DNA damage compared with wild-type (N2) animals. This is accomplished by coordinately inhibiting both NDK-1, a positive regulator of MAPK signalling, and the BH3-only pro-apoptotic protein EGL-1. The dual role for this miR family in antagonizing EGL-1 and NDK-1 reveals a failsafe mechanism to ensure the appropriate magnitude of signalling required for eliminating germ cells in response to genotoxic stress.

## Results

### *mir-35(gk262)* mutants have excessive germ cell death and MAPK activation in response to IR

**Fig. 1:**
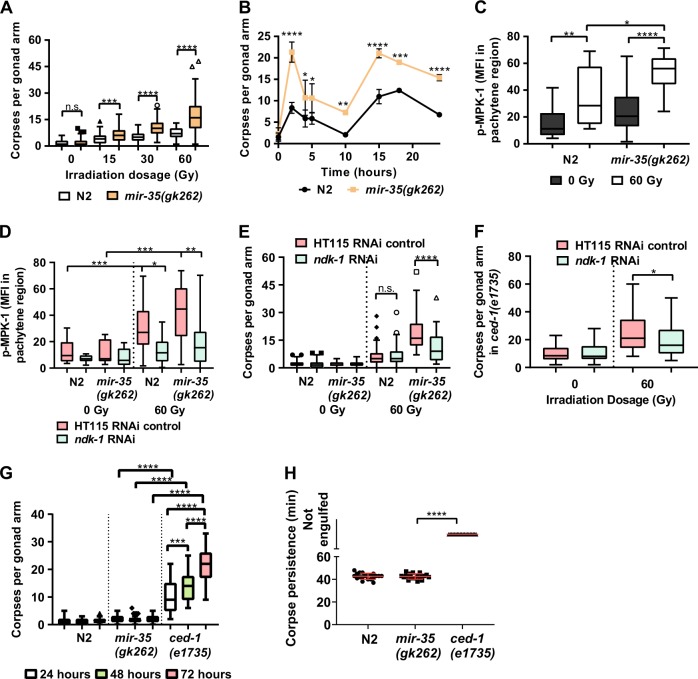
Inappropriate activity of NDK-1 in *mir-35(gk262)* mutant result in increased MAPK activity and germ cell death after exposure to genotoxic stress. Number of corpses at **a** different irradiation dosages in N2 and *mir-35-41(gk262)* mutant (*P* < 0.001 (****), *P* = 0.0002 (***), n.s. = not significant) and **b** at 60 Gy for the indicated times (*P* < 0.0001 (****), *P* = 0.0005 (***), *P* = 0.0076 (**), *P* < 0.01 (*)). Mean fluorescence intensity (MFI) of the pachytene region was determined in isolated germlines stained for p-MPK-1 **c** without RNAi treatment (*P* < 0.0001 (****), *P* = 0.0025 (**), *P* = 0.01 (*)) or **d** with RNAi treatment (*P* < 0.0004 (***), *P* = 0.0012 (**), *P* = 0.01 (*)). Refer to Supplementary Fig 1 and 2, respectively, for representative images used in quantifying p-MPK-1. **e** Corpses numbers in N2 and *mr-35(gk262)* worms fed with the indicated RNAi and irradiated at young adult (*P* < 0.0001 (****), n.s. = not significant). **f** *ced-1(e1735*) on the indicated RNAi and irradiated at young adult (*P* = 0.0174 (*)). Corpses were determined 24 h after irradiation (*n* ≥ 30 worms in three independent replicates). **g** Germ cell corpses and **h** cell corpse persistence was determined in unirradiated N2, *mir-*35(*gk262*) and *ced-1(e1735*) worms at 24, 48 and 72 h post-L4. *P* < 0.0001 (****), *P* = 0.0007 (***). Graphs are plotted as whisker and box plot to illustrate data distribution with symbols indicating outliers based on Tukey’s test. Part (**b**) is a line plot with error bars indicating SEM. Part (**h**) is a scatter plot with errors, illustrated in red for visual ease, indicating SEM

To determine the effect of genotoxic stress on the *C. elegans* germline in the absence of miR-35 activity, we exposed *mir-35(gk262)* mutants to increasing doses of ionizing irradiation (IR) and quantified germ cell corpses. In the absence of IR stress there was no difference in the number of germ cell corpses compared with wild-type Bristol N2. However, at 15 Gy of IR we observed a mean of 11.4 corpses per gonad arm in the *mir-35-41* mutants compared with 2.4 in N2 (Fig. 1a, *P* = 0.0002). Apoptosis increased with IR dosage in both *mir-35(gk262)* and N2, but at all doses *mir-35(gk262)* had ~4-fold more germ cell corpses (Fig. 1a and Supplementary Fig. 1). Unirradiated *mir-35* mutants and N2 worms had similar numbers of corpses, due to normal physiological cell death that occurs independently of *egl-1* (ref. [3]). The hypersensitivity of *mir-35(gk262)* to DNA damage-induced cell death prompted us to ask if apoptosis occurs at earlier time points in these mutants after exposure to IR. Cell death was observed to occur in an oscillating pattern with peaks at 2 h and 15–18 h post-IR, with *mir-35* mutants having higher numbers of corpses than N2 throughout (Fig. 1b; *P* < 0.02).

Ras/MAPK signalling is required for germline development as well as physiological [3] and DNA damage-induced apoptosis [9–12]. When MAPK signalling is reduced, as determined by phosphorylation of the ERK1 orthologue MPK-1, the germline develops but apoptosis is suppressed [3, 9, 12]. Phospho-MPK-1 in N2 germlines displays a distinct pattern of enrichment in early to mid-pachytene region and in oocytes, but absent in the late pachytene/early diplotene region [28–30]. However, p-MPK-1 increases in the late pachytene/early diplotene region in wild-type germlines following irradiation [10]. To evaluate the phosphorylation of MPK-1 in *mir-35(gk262)* mutants we dissected and stained their germlines with an antibody to diphosphorylated ERK-1, which cross-reacts with phosphorylated MPK-1 (ref. [31]). Quantification of fluorescence intensity revealed low levels of p-MPK-1 in the pachytene region in absence of irradiation for both N2 and *mir-35* mutants, but *mir-35* mutants had higher levels of p-MPK-1, even in absence of IR (Supplementary Fig. 2). Although phosphorylation increased significantly after exposure to 60 Gy of IR in both N2 and *mir-35* mutants (Fig. 1c, *P* = 0.0025, *P* < 0.0001, respectively), *mir-35* mutants had higher signal within the pachytene region at 60 Gy (*P* = 0.012).

### Mir-35 targets *ndk-1* to attenuate MAPK activation

To determine how miR-35 regulates the MPK-1/MAPK pathway, we used TargetScan and identified the 3′-UTR of nucleoside disphosphate kinase (*ndk-1*) as a potential target of the *mir-35-42* family. NDK-1 was reported to be a positive regulator of Ras/MAPK signalling in vulva development [32], so we wondered if increased NDK-1 activity in the germline might be responsible for elevated p-MPK-1 in *mir-35* mutants. Ablation of *ndk-1* resulted in no difference in p-MPK-1 compared with controls, but a twofold reduction in p-MPK-1 was observed in *mir-35* mutants after ablation of *ndk-1* (Fig. 1d, *P* < 0.0001, Supplementary Fig. 3). Ablation of NDK-1 did not affect physiological germ cell apoptosis but suppressed IR-induced apoptosis in *mir-35* mutants by half (Fig. 1e, *P* < 0.0001). Interestingly, IR-induced apoptosis in N2 germlines was unaffected by *ndk-1* knockdown, suggesting that *mir-35* normally antagonizes NDK-1 to attenuate MAPK signalling in the germline in response to genotoxic stress.

While NDK-1 was previously reported to regulate engulfment [33], we did not observe an accumulation of corpses after *ndk-1* knockdown in absence of IR (Fig. 1e). On the contrary, we observed a slight decrease in germ corpses in *ced-1(e1735*) mutants on *ndk-1* RNAi (Fig. 1f). Moreover, we did not observe an accumulation of corpses or engulfment rates in *mir-35*(*gk262*) mutants (Fig. 1g, h).

### miR-35 modestly induces deadenylation of the *ndk-1* 3′-UTR

**Fig. 2:**
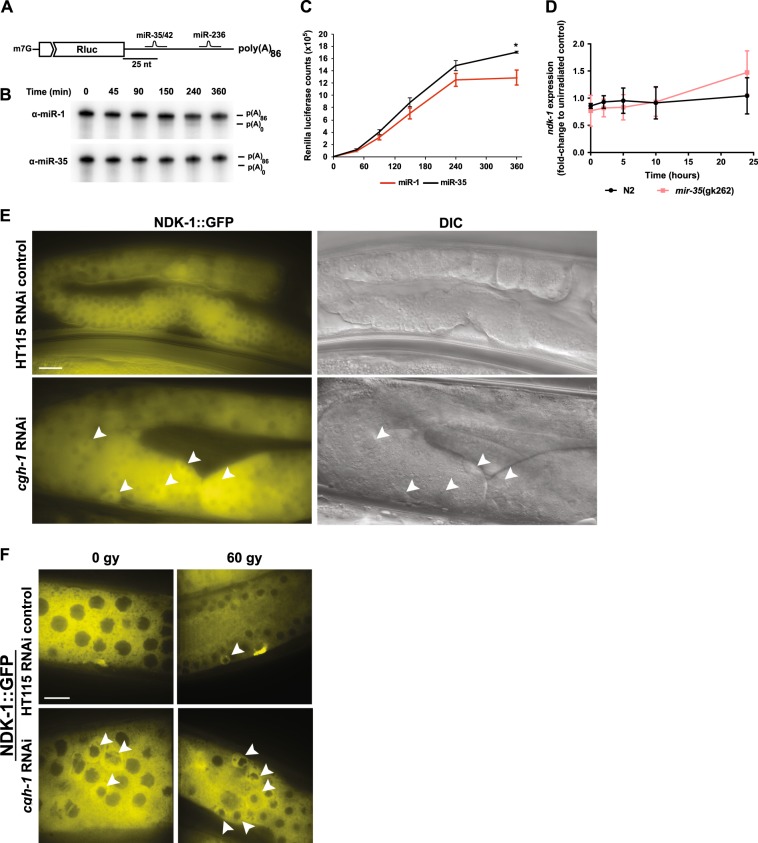
miR-35 inhibits *ndk-1* mRNA translation potentially through association with P-granule CGH-1 and subtle deadenylation activity. **a** Design for the RNA used in the experiment. A capped transcript containing a Renilla luciferase ORF and *ndk-1* 3′-UTR. Predicted target sites for miRNAs expressed in *C. elegans* embryo are shown in black. The reporter was subjected to an in vitro **b** deadenylation assay and **c** translation assay in the presence of a miR-35 or non-specific miR-1 2′-O-Me inhibitor. Translation activity was monitored through measurement of Renilla luciferase activity. **d** Real time PCR of *ndk-1* in N2 and *mir-35(gk262)* young adults at 60 Gy for the indicated time points (three independent replicates). *P* = 0.038 (*), n.s. = not significant. Error bars indicate SEM. L1-staged *ndk-1*::GFP worms were fed HT115 control RNAi or *cgh-1* RNAi. **e** Germlines of unirradiated young adult worm were imaged under ×60 oil objective. White bar represents 100 μm scale. **f** Sections of pachytene region near the loop end of the germline were imaged with a spinning disc confocal under 60X oil objective for unirradiated (0 Gy) or irradiated (60 Gy) young adult worms. Scale bar is 15 μm. GFP was pseudocoloured to yellow for better visualization of signal in CYMK format

Numerous studies have shown that miRNAs post-transcriptionally regulate gene expression by stimulating deadenylation of mRNA 3′-UTR, which leads to the destabilization and degradation of the transcript [23–40]. Indeed, MiR-35 induces deadenylation and degradation of *egl-1* in cooperation with two other miRNAs (miR-58 and miR-80) [23]. To determine if miR-35-stimulates deadenylation of *ndk-1*, we fused the *ndk-1* 3′-UTR to a Renilla luciferase coding sequence and subjected it to an in vitro deadenylation and translational repression assay in the presence of either a miR-35, or non-specific miR-1 2′-O-Me inhibitor (Fig. 2a). Low levels of deadenylation (p(A)_0_ labelled band) were observed in the presence of the non-specific miR-1 inhibitor, whereas the addition of the miR-35 inhibitor abrogated deadenylation (absence of p(A)_0_ labelled band, Fig. 2b) (Fig. 2b, c). However, miR-35-dependent deadenylation of the *ndk-1* 3′-UTR displayed slower kinetics compared with the *egl-1* 3′-UTR [23], suggesting that deadenylation and consequent destabilization of *ndk-1* mRNA transcript is not the mechanism by which miR-35 regulated NDK-1. To test this hypothesis, we measured luciferase activity of the same *ndk-1* reporter and found a significant increase in translation activity upon addition of the miR-35 inhibitor compared with the miR-1 control (Fig. 2c). Real-time PCR performed on whole *mir-35-41(gk262)* mutant animals after irradiation confirmed that *ndk-1* transcript levels remain comparable with wild-type animals (Fig. 2d). Since the in vitro deadenylation and translational inhibition assay was conducted with embryo extracts, it is possible that regulation of miRNAs in the germline may be different than in the embryos, as recently described [41]. Moreover, the short 3′-UTR of *ndk-1* is predicted to have only the miR-35 binding site; therefore, unlike the cooperative activity of multiple miRNAs as in the case of *egl-1*, the single activity of miR-35 on *ndk-1* results in a different mode of regulation.

### Downregulation of P-granule associated CGH-1 protein increases NDK-1 expression

One potential mechanism for translational repression of transcripts in lieu of mRNA degradation is by sequestering them in RNA granules [42]. The *C. elegans* germline has a distinct RNA compartment called P-granules, which are a class of perinuclear RNA granules specific to germ cells that are enriched in the Argonaute family of RNA regulators [43]. In the absence of significant changes in *ndk-1* mRNA, we asked if translational inhibition of *ndk-1* by miR-35 involves RNA compartments in the germline. Therefore, we fed L1-staged *ndk-1*::GFP worms with RNAi to *cgh-1*, which encodes an RNA helicase that localizes to germline P-granules [44] and observed an increase in the number of germ cells with intense GFP signal. This corresponds to phenotypic changes (button shape) of cells undergoing apoptosis (Fig. 2e). There was also a clear increase in germ cells with high GFP expression after 60 Gy IR (Fig. 2f), suggesting that CGH-1 may have a role in miR-35 translational silencing of *ndk-1* in P-granules.

### NDK-1 expression increases prior to cell death

**Fig. 3:**
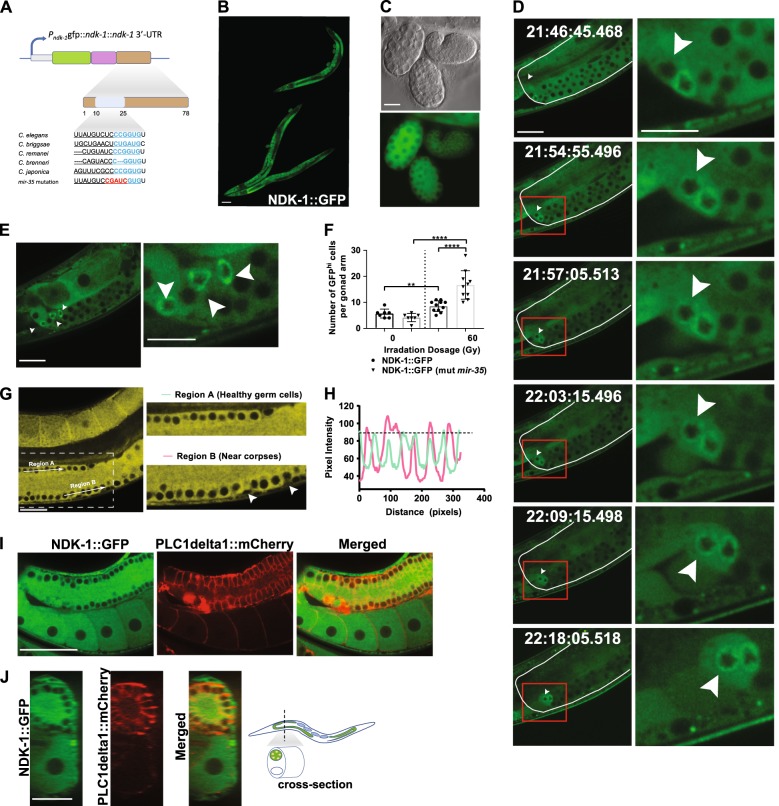
Intense NDK-1 expression precedes cell death in individual germ cells. **a** Schematic diagram of the endogenous *ndk-1* locus with predicted miR-35 binding site in the *ndk-1* 3′-UTR; seed site is indicated in cyan and mutation is indicated in red. **b** Whole worms and **c** embryos expressing GFP tagged NDK-1 in the germline taken at ×20 and ×40 magnification with 100 μm scale bar. **d** Time lapse images and **e** still image of *ndk-1* (*mir-35* mut) strain irradiated at 60 Gy and immediately imaged after irradiation at ×40 water immersion. Regions indicated within red squares in (**d**) are shown as enlarged insets below each image. In **e**, enlarged image of GFP high cells is shown on the right. **f** Cell with highly expressed NDK-1::GFP were quantitated in the adjacent graph (*n* ≥ 7 germlines in three independent replicates, *P* < 0.0001 (****), *P* = 0.0087 (**)). White arrows indicate cell that is about to die showing dramatic increase in GFP intensity. **g**, **h** GFP fluorescence intensity measured in regions around heathy germ cells or corpse bodies. Arrows in inset to the right indicates corpses. GFP was pseudocoloured to yellow for better visualization of signal in CYMK format. **i** Confocal image taken at ×40 magnification in 1 μm Z-stack of NDK-1::GFP; OD70 strain. Germline membrane marked with mCherry. Scale bar is 50 μm. **i** Shows image of germline through worm midline. **j** Shows cross-section of worm as indicated in the diagram. Scale bar is 20 μm

To determine NDK-1 is expressed we tagged the endogenous gene with GFP using CRISPR gene editing (Fig. 3a) and observed robust expression throughout the entire germline (Fig. 3b) and in early embryos (Fig. 3c). This is in contrast to a previous report that observed the expression of an NDK-1::GFP reporter solely in the distal tip cell (DTC) and somatic gonadal sheath cells [33]. Increasing evidence indicates that death signals must overcome a threshold for a cell to undergo apoptosis [45–47]. We observed a dramatic increase in perinuclear GFP::NDK-1 localization in germ cells, minutes before they exhibited the refractile morphology characteristic of apoptosis (Fig. 3d, Supplementary Movies 1 and 2, and Supplementary Fig. 4). The time from the first detection of increased GFP signal to peak fluorescence intensity and cell morphology changes was ~16.83 min (Fig. 3d, Supplementary Movie 1) and ~ 8.15 min (Supplementary Movie 2), respectively. Cells with substantial bursts of GFP intensity were observed solely within the late pachytene region of the germline (Fig. 3e). We observed significantly more bright GFP-positive cells in strains with mutated *mir-35* binding sites in the *ndk-1* 3′-UTR compared with wild type (16.7 vs 8.5 cells per gonad arm, respectively; *P* < 0.0001, Fig. 3f). In addition, there was relatively higher GFP signal in cells near late-stage germ cell corpses (region B, Fig. 3g). Fluorescence intensity was determined along the length of the white arrow, which was drawn across the centre of each germ cell. The troughs indicate absence of GFP signal in the nucleus and the peaks are GFP fluorescence intensity in the cytoplasm. Cells nearest to corpses (white arrows) showed the highest cytoplasmic fluorescence intensity (Fig. 3h), which was not observed in a region surrounding healthy germ cells (region A). *C elegans* germ cells share a common cytoplasm through openings to the rachis [48], but it is still not clear how the live-or-die decisions of a cell affects its neighbours. To pinpoint NDK-1 subcellular localization, we crossed GFP::NDK-1 worms to a strain expressing a germline membrane marker and observed GFP::NDK-1 restricted to the germ cell cytoplasm (Fig. 3i, j). Cells with the highest NDK-1 levels around their nuclei are more prone to undergo apoptosis.

### *egl-1* is post-transcriptionally regulated by the *mir-35* family in the germline

**Fig. 4:**
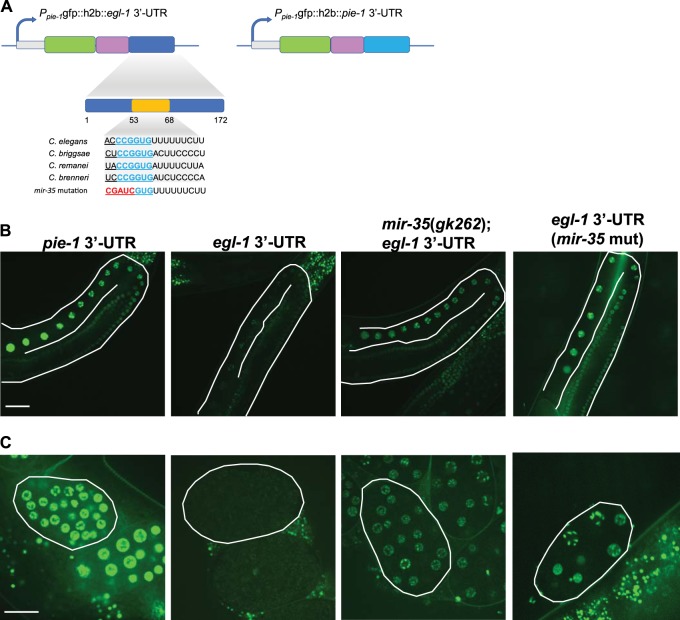
miR-35 targets the *egl-1* 3′-UTR to suppress transcript translation. **a** Schematic diagram of the vector construct with predicted miR-35 binding site in the *egl-1* 3′-UTR; seed site is indicated in cyan and mutation is indicated in red. These vectors were injected into N2 background to create GFP reporter strains containing either *pie-1* 3′-UTR or *egl-1* 3′-UTR under a *pie-1* promoter. The strains were crossed into *mir-35(gk262)* and additionally the miR-35 binding site was mutated in the *egl-1* 3′-UTR containing reporter strain using CRISPR. **b** Germlines and **c** embryos of these strains expressing the GFP reporter were imaged with no irradiation at young adult stage. Representative images are shown here. Images were taken with a water immersion objective at ×40 magnification on Olympus IX81 Quorum spinning disk confocal. Scale bar is 15 μm

Both miRNA-target prediction algorithms TargetScan [49] and MirWIP [50] listed *egl-1* as a potential target for the *mir-35-42* family (Fig. 4a). In addition, Wu et al. reported the deadenylation of the *egl-1* 3′-UTR by miR-35 destabilizes its transcript [23]. To determine if *egl-1* transcript is targeted for post-transcriptional regulation by *mir-35-42* family members in vivo, we cloned the *egl-1* 3′-UTR downstream of a GFP reporter fused to histone H2B under control of the germline-specific *pie-1* promoter [51]. As a control, we constructed the same reporter with a *pie-1* 3′-UTR, which is only 88 bp in length and has only two predicted miRNA binding sites; none of which binds *mir-35-42* family. We observed strong H2B::GFP signal in the nucleus of germ cells, oocytes (Fig. 4b, far left) and embryos (Fig. 4c) for the *pie-1* 3′-UTR control reporter. However, GFP signal was greatly reduced in the germlines and embryos of worms expressing the *egl-1* 3′-UTR reporter (Fig. 4b, centre left), suggesting factors in the germline and embryos inhibit translation through the *egl-1* 3′-UTR. We next wanted to determine if binding of the *mir-35* family of miRNAs to predicted seed sequences on the *egl-1* 3′-UTR promotes this negative regulation. Therefore, we introduced the GFP reporter with the *egl-1* 3′-UTR into *mir-35(gk262)* mutants and observed a striking increase in GFP in germ cells, oocytes and embryos (Fig. 4b, c, centre right). To ensure that de-repression of this reporter was specific to miR-35 activity, we mutated the miRNA seed binding site in the *egl-1* 3′-UTR (Fig. 4a), which resulted in a comparable increase in GFP (Fig. 4b, c, far right). Noticeably, the GFP signal did not reach the same intensity as observed with the *pie-1* 3′-UTR, suggesting that other inhibitory factors might cooperate with miR-35.

### miR-35 buffers *egl-1* expression after exposure to genotoxic stress

**Fig. 5:**
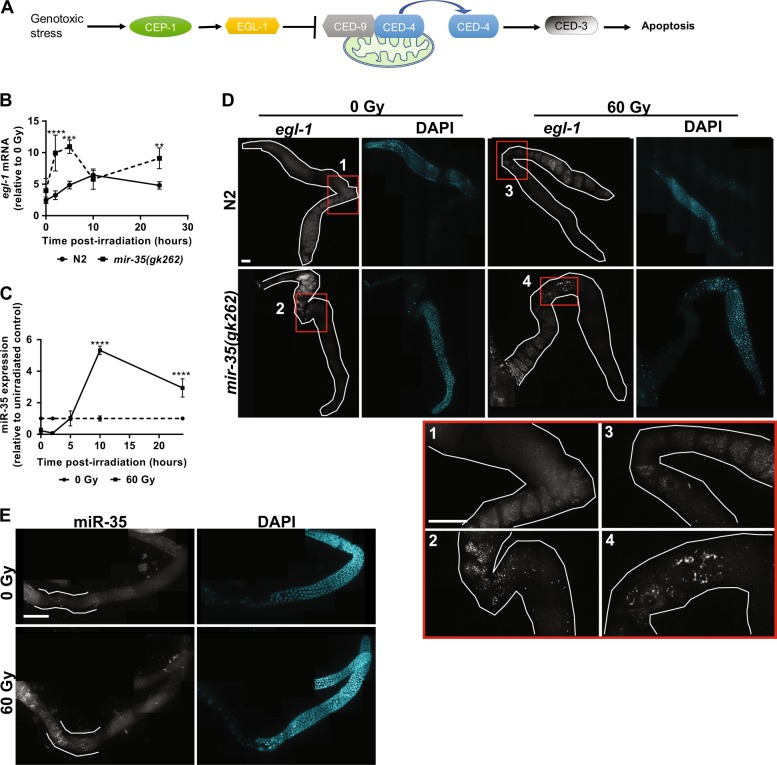
Increased *egl-1 mRNA* and miR-35 expression is restricted to the pachytene region of the germline after genotoxic stress. **a** Schematic diagram of the core apoptosis pathway. **b** Real time PCR for *egl-1* transcripts at 60 Gy at the indicated times (*P* < 0.0001 (****), *P* = 0.0002 (***), *P* = 0.0075 (**). **c** Real-time PCR for miR-35 at 60 Gy at the indicated times in N2 (*n* ≥ 30 worms in three independent replicates, (*P* < 0.0001 (****)). **d** *egl-1* mRNA FISH probe in N2 and *mir-35(gk262)* germlines and **e** miR-35 LNA-smFISH probe in isolated N2 germlines of young adult worms irradiated at 0 or 60 Gy. Germlines were isolated at 2 h post-irradiation for *egl-1* mRNA and 5 h post-irradiation for miR-35. Regions indicated within red squares are shown as enlarged insets numbered 1–4 in panel (**d**). Confocal images were taken at ×60 oil, Z-stack at 1 μm slices and germline images were stitched together. Error bars indicate SEM. Scale bar is 34 μm

In the *C. elegans* germline, genotoxic stress causes double-strand breaks that activate the DNA damage checkpoint and CEP-1 to induce EGL-1 expression and trigger apoptosis (Fig. 5a). In the absence of *mir-35-41* we observed two peaks of *egl-1* mRNA at 2–5 h (*P* < 0.0002) and 24 h (*P* = 0.0075) after irradiation (Fig. 5b). Since miRNA levels fluctuate to buffer excessive noise in gene expression [52], we asked if *mir-35* levels also increase after irradiation. Thus, we exposed N2 worms to 60 Gy IR then isolated total RNA at the indicated time points and quantified miR-35 expression by real-time PCR. miR-35 expression was highest 10 h post-irradiation, with ~ 5-fold greater expression relative to unirradiated worms (Fig. 5c, *P* < 0.0001). We also quantified expression levels of each *mir-35* miRNA family member after 24 h with or without irradiation and observed significantly increased expression of miR-35, -36, -40, -41 and -42 family members, suggesting that they may have a more prominent roles in regulating *egl-1* after irradiation (Supplementary Fig. 5).

### *egl-1* and miR-35 mRNA accumulate in the pachytene germline after irradiation

Since germ cell death is only observed shortly before the cells exit the pachytene phase of prophase I [3], we asked if *egl-1* transcript accumulates in this region after irradiation. Since *egl-1* is a target of miR-35 we also wondered if miR-35 was enriched in the same region. LNA-FISH probes to *egl-1* revealed accumulation of transcripts within the pachytene region after genotoxic stress compared with unirradiated controls in both N2 and *mir-35* mutants (Fig. 5d and Supplementary Movie 3a–d). In addition, *egl-1* transcripts accumulated into large patches within the pachytene region of the *mir-35* mutants compared with N2, and there was greater accumulation of *miR-35* within the same proximity in germlines exposed to 60 Gy (Fig. 5e). *mir-35(gk262)* mutants proceed normally through all stages of germline development, including progression through the mitotic zone, transition zone, pachytene, diplotene and diakinesis (Supplementary Fig. 6). Thus, the increase in cell death and localization of miR-35 expression is not a consequence of aberrant germline development.

### EGL-1 and NDK-1 likely function in the same pathway

**Fig. 6:**
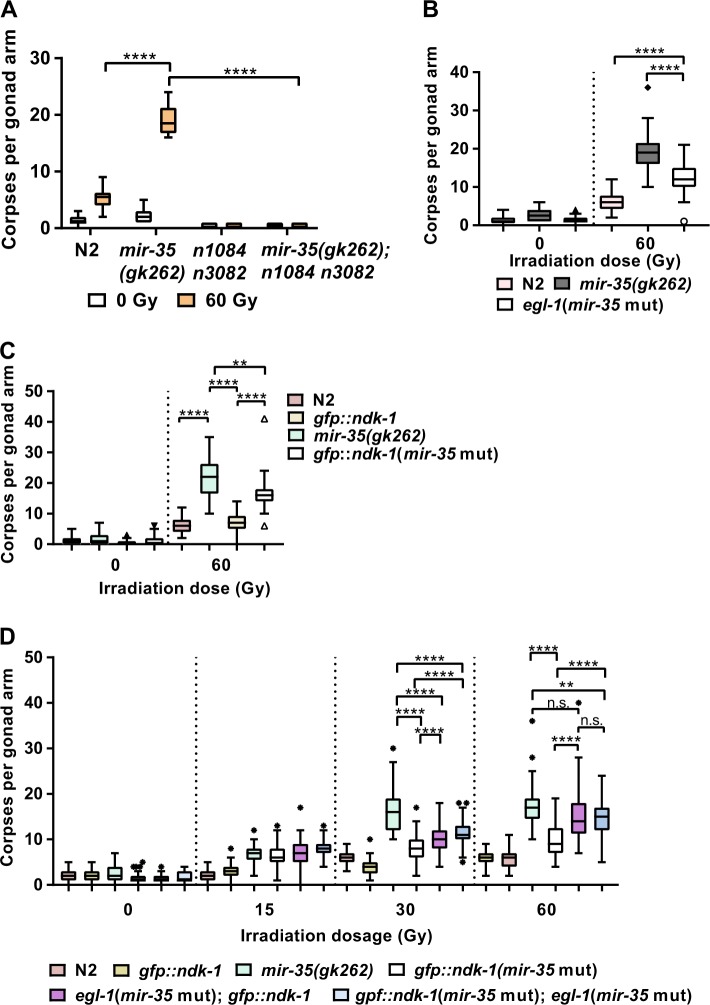
miR-35 inhibits extraneous germ cell death by negatively regulating both *egl-1* and *ndk-1* expression. **a** Cell death is inhibited in *mir-35(gk262)*; *egl-1(n1084 n3082)* loss-of-function double mutant (*P* < 0.0001 (****)). Worms were irradiated at young adult stage and corpses counted 24 h after irradiation. Number of corpses in **b** *egl-1* 3′-UTR (miR-35 mutant) (*P* < 0.0001 (****)) or **c** *ndk-1* 3′-UTR (*mir-35* mutant) (*P* < 0.0001 (****), *P* = 0.0017 (**)) or **d** in the double mutant *egl-1* 3′-UTR (*mir-35* mut.); *ndk-1* 3′-UTR (miR-35 mutant) young adults at 0 or 60 Gy (*P* < 0.0001 (****), *P* = 0.003 (**), n.s. = not significant). Worms were irradiated at young adult stage and corpses counted 24 h after irradiation. Symbols indicate outliers based on Tukey’s test (*n* ≥ 45 worms in three independent replicates)

To determine if NDK-1 and EGL-1 act in the same or parallel pathways, we quantified germ cell apoptosis of *egl-1* and *ndk-1* miR-35 binding site double mutants (Figs. 3a and  4a). The *egl-1* miR-35 binding site mutant was generated using CRISPR/Cas9 to introduce the same mutation depicted in Fig. 4a into the endogenous *egl-1* 3′-UTR locus. Mutation of the *mir-35* site in the *egl-1* 3′-UTR resulted in corpse levels comparable with those observed in *mir-35(gk262)* deletion mutants (Fig. 6b). Similarly, mutation of *mir-35* seed site in the *ndk-1* 3′-UTR increased cell death to approximately half the level observed in *mir-35(gk262)* mutants (Fig. 6c). When both *mir-35* binding sites were mutated in the same strain the number of corpses detected was similar the *egl-1* 3′-UTR *mir-35* single mutant (Fig. 6d). This suggests that *egl-1* acts downstream of *ndk-1* in the same pathway.

## Discussion

Spatial and temporal regulation of the apoptotic response in the germline is distinct from the soma because this syncytium shares a common cytoplasm and many nuclei can be exposed to factors secreted from dying (and living) cells. This poses the challenge of constraining DNA damage-induced death of cells to only those with damage beyond repair. One major question concerns the mechanisms utilized to prevent inappropriate death of these cells. Recent work by Sherrard et al. provided some important insights, where microRNAs *mir-35* and *mir-58* promote survival of sister cells in somatic lineages of the *C. elegans* embryo by targeting *egl-1* transcripts [27]. However, embryonic cells in this organism do not exist in a syncytium, are fated to die during specific stages of development, and do not undergo apoptosis in response to stress. Therefore, we wondered if different regulatory mechanisms were involved in the control of germline apoptosis through *egl-1*.

Indeed, we found a dual function for miR-35 in regulating both the MAPK and the core apoptotic pathways by antagonizing *egl-1* and *ndk-1*. The *mir-35(gk262)* deletion allele hypersensitizes germ cells to apoptosis induced by genotoxic stress. This was completely abolished by the *egl-1* loss-of-function allele *egl-1(n1084 n3082)*, but mutating the *mir-35-*binding site in the *egl-1* 3′-UTR resulted in enhanced levels of apoptosis comparable with *mir-35(gk262)* mutants. This indicates that increased levels of apoptosis in response to IR was in part due to the lack of miR-35 regulation of EGL-1. The increased phosphorylation of ERK1/2 orthologue MPK-1 in *mir-35* mutants prompted us to ask if miR-35 might also antagonize the MAPK pathway. We identified a miR-35-binding site in *ndk-1*, which encodes the only orthologue of human NME1 nucleoside diphosphate kinase family in *C. elegans* [53]. NME1 is a metastasis suppressor of murine melanoma cells [54] and a broad spectrum of human tumours including breast, thyroid and gastric cancers [55, 56]. NME1 also has an orthologue in *Drosophila* called *abnormal wing discs* (*awd*), which induces imaginal disc cell death [57] and epithelial tubule morphogenesis in the trachea [58]. Human NME1 has also been suggested to inhibit Ras/ERK signalling [59–62]. In contrast, the *C. elegans* NDK-1 is reported to be a positive regulator of Ras/MAPK signalling during vulva development, which functions downstream of LIN-45/Raf and upstream of MEK-2/MEK and MPK-1/MAPK, potentially through the worm kinase suppressor of Ras gene *ksr-2* (ref. [32]). Although a previous study reported NDK-1 expression in the DTC and gonadal sheath cells [33], we observed cytoplasmic expression throughout the germline using a reporter tagged at the endogenous locus.

We found that elevated MPK-1 phosphorylation (and apoptotic bodies) in the pachytene region of *mir-35* mutants was the result of increased NDK-1 activity, indicating it can stimulate MPK-1-dependent apoptosis in the germline. Since increased MPK-1 phosphorylation was evident in both the absence and presence of IR, but there was no increase in physiological apoptosis, we suggest that the miR-35 family acts to buffer MAPK activity under normal and stressed conditions. Mammalian NME1 was reported to phosphorylate the KSR1 scaffold of the MAPK pathway kinases [60, 62]; however, the specific function of KSR-2 and the substrate(s) of NDK-1 in the worm germline remain to be defined. Our results suggest that MAPK signalling in the germline is negatively regulated by miR-35 through post-transcriptional control of NDK-1, which when hyperactivated stimulates the core cell death pathway.

When the *mir-35* binding site was mutated in the *ndk-1* 3′-UTR apoptosis increased to about half the level observed in *mir-35(gk262)* mutants, whereas mutation of the *mir-35* site in the *egl-1* 3′-UTR resulted in comparable levels of apoptotic corpses as seen in *mir-35* mutants. While this suggests that additional factors regulate apoptosis upstream of *egl-1*, *ndk-1* likely functions in the same pathway as *egl-1* which is consistent with corpse numbers when miR-35 binding sites are mutated in both *egl-1* and *ndk-1*. Since BH3-only pro-apoptotic proteins is known to be regulated by phosphorylation in mammals [63, 64], EGL-1 may be subjected to post-translational regulation by MAPK signalling, or directly by NDK-1.

One mechanism by which miRNAs regulate protein expression is through deadenylation of the 3′-UTR of transcripts, which leads to destabilization and degradation of the mRNA. miR-35 was previously shown to cause the deadenylation of the *egl-1* 3′-UTR [23], but in this study we show binding to the *ndk-1* 3′-UTR has a very subtle effect on *ndk-1* 3′-UTR deadenylation compared with *egl-1* (ref. [23]). Since there was little change in *ndk-1* transcript in *mir-35* mutants after irradiation, we suggest that miR-35 may regulate NDK-1 through translational repression [65, 66], possibly by sequestering it into P-bodies [42]. *C. elegans* has been reported to contain two distinct RNA compartments called processing bodies (P-bodies) and P-granules [67, 68]. Untranslated mRNAs accumulate in cytoplasmic P-bodies in somatic cells where specific protein complexes inhibit translation and stimulate mRNA decay. On the other hand, P-granules are a class of perinuclear RNA-containing structures specific to germ cells that are enriched in the Argonaute family of RNA regulators [43]. We showed that CGH-1, an RNA helicase known to localize to P-granules [69, 70], is involved in miR-35 translational inhibition of *ndk-1* mRNA. Navarro et al. found that CGH-1 is specifically expressed in *C. elegans* germline and early embryo, and localizes to P-granules and other cytoplasmic mRNA-protein foci [44]. Inhibition of CGH-1 activity resulted in excessive physiological germ cell death. Since a certain basal level of NDK-1 is required for proper development of the germline [32], dynamic changes to transcript levels may be detrimental to the germline. Thus, translational repression and release with stimuli such as DNA damage may be a more efficient mechanism to regulate NDK-1 and fine-tune the apoptotic response.

**Fig. 7:**
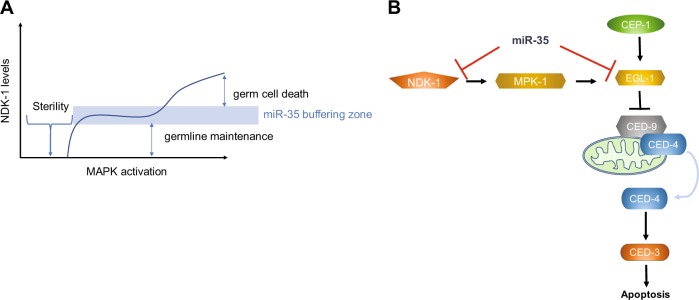
miR-35 regulates germ cell death by buffering MAPK activity via negative regulation of *ndk-1* and by also targeting *egl-1* after genotoxic stress. **a** miR-35 acts to buffer NDK-1 activation of MAPK. Absence of miR-35 result in more cells enriched in NDK-1 protein, which leads to increase in MAPK activity and germ cell death. **b** Schematic diagram of model for miR-35 inhibition of MAPK and core apoptosis pathway activation. MiR-35 negatively regulates germ cell death by targeting both EGL-1 in the core apoptosis pathway and NDK-1, a positive regulator of MAPK signalling

The rapid increase in perinuclear localization of NDK-1 in germ cells prior to apoptosis suggests a role for miR-35 in controlling NDK-1 translation (or transcript localization) to fully commit a cell to death. This intense NDK-1 localization may also serve as an early marker of cell death in the *C. elegans* germline. Based on these observations, we propose that miR-35 serves to simultaneously antagonize the hyperactivation of both EGL-1 and NDK-1 to prevent massive levels of apoptosis after genotoxic stress (Fig. 7a). Furthermore, epistasis analysis suggests that NDK-1 functions upstream, or at the level, of EGL-1 to potentiate the apoptotic response (Fig. 7b). The continuous and repeated activation of the death signalling pathways, resulting from persistent DNA lesions, may be necessary to stimulate a positive regulatory factor to overcome the apoptotic threshold. Thus, combined activation of MAPK, via its positive regulator NDK-1, and EGL-1 may explain how a careful balance of these two arms ensure the cell overcomes the apoptotic threshold. On the other hand, miR-35 may establish a buffering zone in neighbouring cells that prevents inappropriate activation of cell death from diffused death signals such as outflow of *egl-1* from dying neighbours. The duality of miRNA in targeting separate but converging pathways may function as a failsafe mechanism to ensure the proper balance of signals that dictate whether a cell lives or dies.

## Materials and methods

### *C. elegans* strains and maintenance

Worms were cultivated on lawns of *Escherichia coli* (strain OP50) grown on nematode growth medium (NGM) plates at 20 °C, unless otherwise stated [71]. The following strains were provided by the *Caenorhabditis* Genetics Centre: VC514, MT8735 and CB3203. WT refers to the *C. elegans* variety Bristol, strain N2. The following strains were developed by crossing VC514 to each of WD366 or WD367, respectively: WD400 and WD408. The *egl-1* null mutation was introduced into the *mir-35(gk262)* mutant background by crossing MT8735 to VC514.

### CRISPR/Cas9, MosSci constructs and microinjection

Introduction of N-terminus GFP tag in the *ndk-1* ORF (WD547) and/or mutation into *mir-35* binding site in the endogenous 3′-UTR of *ndk-1* (WD566) or *egl-1* (WD623) was done by CRISPR/CAS9 as previously described [72]. pDD282 containing *ndk-1* homology arms and N-terminal GFP tag was co-injected with pCFJ90 (P_*myo-2*_::mCherry) and pGH8 (P_*rab-3*_::mCherry) into N2 young adult worms. The *egl-1* 3′-UTR or *pie-1* 3′-UTR was cloned into P_*pie-1*_::GFP::H2B MosSci vector and co-injected with pCFJ90 and pGH8 into N2 young adult worms. Mutations in miR-35 binding site was introduced into the MoSci vector using the Q5 site-directed mutagenesis kit (NEB) according to manufacturer’s instruction. Microinjection was performed using a FemtoJet (Eppendorf) microinjection system attached to an inverted Leica DMI3000B microscope. See Supplementary Table 1 for the primers list.

### Quantification of germ cell apoptosis

Worms were picked at the L4 stage, aged to young adults for 24 h, and then subjected to gamma-irradiation from a ^137^Cs source. After irradiation, all worms were incubated at 20 °C for 24 h (unless otherwise mentioned) before apoptotic cells were enumerated. Worms were immobilized in ~ 20 µl of 20 mM L-tetramisole in M9 buffer on a 4% agarose pad on a glass slide. Apoptotic cells were counted under ×60 oil objective on a Leica DMRA2 compound microscope (Wetzlar, Germany) using standard differential interference contrast (DIC) optics according to Lant and Derry [73]. Images of worm germlines were captured with a Hamamatsu C472-95 digital camera using OpenLab software (PerkinElmer Inc.).

### Real-time PCR

Total mRNA was isolated using TRIzol® Plus RNA Purification Kit (Ambion, Life Technologies). Briefly, worms were transferred to 1 ml of TRIzol®, flash freeze in dry ice/ethanol mixture for 30 min. Then 200 ml chloroform was added to thawed samples and vortexed for 15 min on high at room temperature. Total mRNA isolation then proceeded as per manufacturer’s protocol. Briefly, 500 ng of purified mRNA was used to generate cDNA with random hexamer primers (Thermo Fisher Scientific) SuperScript^®^III Reverse Transcriptase (Invitrogen, Life Technologies) according to manufacturer’s protocol for mRNA, and real-time PCR reaction for miRNA was carried out as previously published [74, 75]. Real-time PCR reaction mix (12.5 ml) consisted of 6.25 µl of SYBR^®^ Green Master Mix (Bio-Rad), 1 µl cDNA template and 0.25 ml of each of 100 μM forward and reverse primers (Supplementary Table 1). Reactions were run in triplicate on Bio-Rad CFX96 Touch™ Real-Time PCR Detection System and analysed with CFX Manager™ Software v3.1. Cq values were normalized to *rrn-1.1* (Accession no. F31C3.7.1) for mRNA or sn2841 (NCBI Accession #AC006654) for miRNA.

### RNA interference

RNA interference (RNAi) was performed by feeding bacteria expressing double-stranded (ds) RNA, from the Source BioScience LifeSciences feeding library, to L1-staged worms (unless otherwise indicated). RNAi bacteria was inoculated in liquid culture (LB media with 100 mg/ml ampicillin and 50 mg/ml tetracycline) and incubated for 24 h at 37 °C in an orbital shaker. The bacteria was subcultured in a 1:2 dilution with LB + ampicillin + tetracycline for 4 h at 37 °C with shaking, prior to induction with isopropyl-β-D-thiogalactoside (IPTG, 0.1 M) for a further 4 h. Induced bacteria was concentrated 10× before plating onto RNAi media plates (containing 1 μM IPTG and 100 μg/ml carbenicillin). Plates were incubated at 37 °C for 18 h. Worms were staged at L1 through hypochlorite bleaching. Overgrown plates (containing many eggs) were treated with hypochlorite bleach for 5 min at room temperature, before having the bleach transferred to Eppendorf tubes. The tubes of bleached worms were centrifuged at 2000 rpm for 1 min and were followed by two more bleach washes/centrifugation steps and three washes/centrifugation with M9 buffer to remove worm carcasses. Eppendorf tubes containing eggs in M9 buffer were incubated at 20°C in a rotary mixer for 24 h, after which L1 stage animals were dispensed onto NGM plates.

### Phospho-ERK staining

Germlines from young adult worms were dissected on glass slides and fixed with 2% paraformaldehyde for 10 min at room temperature. Fixed germlines were freeze cracked on dry ice and immersed in 100% methanol for 5 min. The germlines were incubated with a 1:1 methanol:acetone solution for 5 min then transferred to 100% acetone for another 5 min. Fixed germlines were washed in 1× PBS/0.1% Tween20 (PBST) four times for 10 min each. The samples were incubated in 1 drop of Image-iT (Thermo Fisher Scientific) for 20 min and blocked in PBST/1% BSA for 1 h. phospho-MPK-1 was probed with monoclonal α-p-ERK antibody (Cell Signalling #4370) and monoclonal α-Nuclear Pore Complex antibody (Mab414, Abcam) in PBST/1% BSA overnight at room temperature. Germlines were washed in PBST three times for 10 min each and probed with goat α-rabbit-conjugated Alexa Fluor 488 (Thermo Fisher Scientific) and donkey α-mouse Alexa Fluor 568 at room temperature for 1 h. Stained germlines were then washed with PBST for 10 min and nuclear DNA was stained with DAPI. Samples were coated with Prolong Gold (Thermo Fisher Scientific) and imaged as described above.

### LNA-FISH

Locked nucleic acid (LNA) probes were designed for *mir-35* (LNA probe: 5′-BioTEG/ACTGC+TAGTT+TCC+ACCCGGTGA, Exiqon). LNA nucleotides are designated with a “+” sign before the letter. A total of 27 Stellaris^®^ FISH probes conjugated with Quasar^®^ 670 Dye were designed for the *egl-1* mRNA (Supplementary Table 1). Germlines were dissected as mentioned above under “ERK staining”. Dissected germlines were fixed with 4% PFA at room temperature for 10 min, then flash freeze on dry ice for 1 h with coverslip. Germlines were freeze cracked by snapping away the coverslip while the sample was still frozen. Germlines were thawed, and 70 ml of EDC solution was added. Slides were incubated at room temperature for 2 h, then washed twice with 1× PBS. Germlines were submerged in 70% ethanol and incubated at 4 °C overnight. The next day samples were washed twice with 1× PBS, then prehybridized with 200 μl prehybridization buffer at room temperature for 15 min. Probes were mixed in hybridization buffer at 125 nM final concentration and added to samples. Coverslips were placed over the germlines and sealed with rubber cement before incubation in a hybridization oven at 60 °C for *mir-35* LNA probe and 30 °C for Stellaris^®^ *egl-1* mRNA probes. Samples were subject to high stringency washes of 5× SSC + 50% formamide, 2× SSC + 50% formamide, 0.2× SSC + 50% formamide and then 0.2× SSC. LNA Probes were detected with secondary antibodies: streptavidin conjugated with Alexa Fluor^®^ 488 (Thermo Fisher Scientific) and anti-DIG antibody conjugated with Alexa Fluor^®^ 567 (Thermo Fisher Scientific). Slides were viewed under an Olympus IX81 Quorum spinning disk confocal with ×60 oil objective and imaged with Hamamatsu C9100-13 EM-CCD camera. Z-stacks were taken with Improvision Piezo focus drive. Images were processed with Perkin Elmer Volocity software and Adobe Photoshop.

### NDK-1::GFP movie

Young adult *ndk-1*::GFP (*mir-35* mut) worms were irradiated at 60 Gy, immobilized on 2% agarose and 1 mM tetramisole, and continuously imaged for 2 h under ×40 water objective with Olympus IX81. For FISH-stained germline imaging was taken with ×60 oil objective. Images were taken at six images per minute with 1 μm Z-stack slices and whole worm image was stitched together with Perkin Elmer Volocity software. In order to keep the target germ cell in the same focal plane for the duration of the movie, specific images were captured to process together to produce the movies in the Supplementary section.

### In vitro deadenylation and translation

In vitro transcription, mRNA stability and target cloning, and deadenylation assays were performed as previously described [23]. Embryonic extracts and in vitro translation assays were carried out as previously published [40].

### Statistical analyses

Statistical significance was determined using Student’s *t*-test, ANOVA and Mann-Whitney test as calculated by GraphPad Prism 7.04.

## Supplementary information

Supplementary Movie 1

Supplementary Movie 2

Supplementary Movie 3A

Supplementary Movie 3B

Supplementary Movie 3C

Supplementary Movie 3D

Supplementary Table 1

Supplementary Figure Legends

Supplementary Figure 1

Supplementary Figure 2

Supplementary Figure 3

Supplementary Figure 4

Supplementary Figure 5

Supplementary Figure 6

## References

[1] Zhang T, Brazhnik P, Tyson JJ (2009). Computational analysis of dynamical responses to the intrinsic pathway of programmed cell death. Biophys J.

[2] Gartner A, Milstein S, Ahmed S, Hodgkin J, Hengartner MO (2000). A conserved checkpoint pathway mediates DNA damage–induced apoptosis and cell cycle arrest in *C. elegans*. Mol Cell.

[3] Gumienny TL, Lambie E, Hartwieg E, Horvitz HR, Hengartner MO (1999). Genetic control of programmed cell death in the Caenorhabditis elegans hermaphrodite germline. Development.

[4] Derry WB, Putzke AP, Rothman JH (2001). Caenorhabditis elegans p53: role in apoptosis, meiosis, and stress resistance. Science.

[5] Schumacher B, Hofmann K, Boulton S, Gartner A (2001). The C. elegans homolog of the p53 tumor suppressor is required for DNA damage-induced apoptosis. Curr Biol.

[6] Schumacher B, Hanazawa M, Lee MH, Nayak S, Volkmann K, Hofmann ER (2005). Translational repression of C. elegans p53 by GLD-1 regulates DNA damage-induced apoptosis. Cell.

[7] Hofmann ER, Milstein S, Boulton SJ, Ye M, Hofmann JJ, Stergiou L (2002). Caenorhabditis elegans HUS-1 is a DNA damage checkpoint protein required for genome stability and EGL-1-mediated apoptosis. Curr Biol.

[8] Conradt B, Wu YC, Xue D (2016). Programmed cell death during Caenorhabditis elegans development. Genetics.

[9] Perrin AJ, Gunda M, Yu B, Yen K, Ito S, Forster S (2013). Noncanonical control of C. elegans germline apoptosis by the insulin/IGF-1 and Ras/MAPK signaling pathways. Cell Death Differ.

[10] Rutkowski R, Dickinson R, Stewart G, Craig A, Schimpl M, Keyse SM (2011). Regulation of Caenorhabditis elegans p53/CEP-1-dependent germ cell apoptosis by Ras/MAPK signaling. PLoS Genet.

[11] Li X, Johnson RW, Park D, Chin-Sang I, Chamberlin HM (2012). Somatic gonad sheath cells and Eph receptor signaling promote germ-cell death in C. elegans. Cell Death Differ.

[12] Eberhard R, Stergiou L, Hofmann ER, Hofmann J, Haenni S, Teo Y (2013). Ribosome synthesis and MAPK activity modulate ionizing radiation-induced germ cell apoptosis in Caenorhabditis elegans. PLoS Genet.

[13] Lima RT, Busacca S, Almeida GM, Gaudino G, Fennell DA, Vasconcelos MH (2011). MicroRNA regulation of core apoptosis pathways in cancer. Eur J Cancer.

[14] Jovanovic M, Hengartner MO (2006). miRNAs and apoptosis: RNAs to die for. Oncogene.

[15] Ishikawa K, Ishikawa A, Shoji Y, Imai T (2014). A genotoxic stress-responsive miRNA, miR-574-3p, delays cell growth by suppressing the enhancer of rudimentary homolog gene in vitro. Int J Mol Sci.

[16] Aalto AP, Pasquinelli AE (2012). Small non-coding RNAs mount a silent revolution in gene expression. Curr Opin Cell Biol.

[17] Friedman RC, Farh KK, Burge CB, Bartel DP (2009). Most mammalian mRNAs are conserved targets of microRNAs. Genome Res.

[18] Bartel DP (2009). MicroRNAs: target recognition and regulatory functions. Cell.

[19] Neilson JR, Zheng GX, Burge CB, Sharp PA (2007). Dynamic regulation of miRNA expression in ordered stages of cellular development. Genes Dev.

[20] Zhang L, Ding L, Cheung TH, Dong MQ, Chen J, Sewell AK (2007). Systematic identification of C. elegans miRISC proteins, miRNAs, and mRNA targets by their interactions with GW182 proteins AIN-1 and AIN-2. Mol Cell.

[21] Ding L, Spencer A, Morita K, Han M (2005). The developmental timing regulator AIN-1 interacts with miRISCs and may target the argonaute protein ALG-1 to cytoplasmic P bodies in C. elegans. Mol Cell.

[22] Lau NC, Lim LP, Weinstein EG, Bartel DP (2001). An abundant class of tiny RNAs with probable regulatory roles in Caenorhabditis elegans. Science.

[23] Wu E, Thivierge C, Flamand M, Mathonnet G, Vashisht AA, Wohlschlegel J (2010). Pervasive and cooperative deadenylation of 3’UTRs by embryonic microRNA families. Mol Cell.

[24] Alvarez-Saavedra E, Horvitz HR (2010). Many families of C. elegans microRNAs are not essential for development or viability. Curr Biol.

[25] Liu M, Liu P, Zhang L, Cai Q, Gao G, Zhang W (2011). mir-35 is involved in intestine cell G1/S transition and germ cell proliferation in C. elegans. Cell Res.

[26] Massirer KB, Perez SG, Mondol V, Pasquinelli AE (2012). The miR-35-41 family of microRNAs regulates RNAi sensitivity in Caenorhabditis elegans. PLoS Genet.

[27] Sherrard R, Luehr S, Holzkamp H, McJunkin K, Memar N, Conradt B (2017). miRNAs cooperate in apoptosis regulation during C. elegans development. Genes Dev.

[28] Lee MH, Ohmachi M, Arur S, Nayak S, Francis R, Church D (2007). Multiple functions and dynamic activation of MPK-1 extracellular signal-regulated kinase signaling in Caenorhabditis elegans germline development. Genetics.

[29] Hajnal A, Berset T (2002). The C.elegans MAPK phosphatase LIP-1 is required for the G(2)/M meiotic arrest of developing oocytes. EMBO J.

[30] Page BD, Guedes S, Waring D, Priess JR (2001). The C. elegans E2F- and DP-related proteins are required for embryonic asymmetry and negatively regulate Ras/MAPK signaling. Mol Cell.

[31] Arur S, Ohmachi M, Berkseth M, Nayak S, Hansen D, Zarkower D (2011). MPK-1 ERK controls membrane organization in C. elegans oogenesis via a sex-determination module. Dev Cell.

[32] Masoudi N, Fancsalszky L, Pourkarimi E, Vellai T, Alexa A, Remenyi A (2013). The NM23-H1/H2 homolog NDK-1 is required for full activation of Ras signaling in C. elegans. Development.

[33] Fancsalszky L, Monostori E, Farkas Z, Pourkarimi E, Masoudi N, Hargitai B (2014). NDK-1, the homolog of NM23-H1/H2 regulates cell migration and apoptotic engulfment in C. elegans. PLoS ONE.

[34] Baek D, Villen J, Shin C, Camargo FD, Gygi SP, Bartel DP (2008). The impact of microRNAs on protein output. Nature.

[35] Bagga S, Bracht J, Hunter S, Massirer K, Holtz J, Eachus R (2005). Regulation by let-7 and lin-4 miRNAs results in target mRNA degradation. Cell.

[36] Behm-Ansmant I, Rehwinkel J, Doerks T, Stark A, Bork P, Izaurralde E (2006). mRNA degradation by miRNAs and GW182 requires both CCR4:NOT deadenylase and DCP1:DCP2 decapping complexes. Genes Dev.

[37] Guo H, Ingolia NT, Weissman JS, Bartel DP (2010). Mammalian microRNAs predominantly act to decrease target mRNA levels. Nature.

[38] Giraldez AJ, Mishima Y, Rihel J, Grocock RJ, Van Dongen S, Inoue K (2006). Zebrafish MiR-430 promotes deadenylation and clearance of maternal mRNAs. Science.

[39] Niinuma S, Fukaya T, Tomari Y (2016). CCR4 and CAF1 deadenylases have an intrinsic activity to remove the post-poly(A) sequence. RNA.

[40] Flamand MN, Wu E, Vashisht A, Jannot G, Keiper BD, Simard MJ (2016). Poly(A)-binding proteins are required for microRNA-mediated silencing and to promote target deadenylation in C. elegans. Nucleic Acids Res.

[41] Dallaire A, Frederick PM, Simard MJ (2018). Somatic and germline MicroRNAs form distinct silencing complexes to regulate their target mRNAs differently. Dev Cell.

[42] Pillai RS (2005). MicroRNA function: multiple mechanisms for a tiny RNA?. RNA.

[43] Shirayama M, Stanney W, Gu W, Seth M, Mello CC (2014). The Vasa Homolog RDE-12 engages target mRNA and multiple argonaute proteins to promote RNAi in C. elegans. Curr Biol.

[44] Navarro RE, Blackwell TK (2005). Requirement for P granules and meiosis for accumulation of the germline RNA helicase CGH-1. Genesis.

[45] Dai H, Ding H, Peterson KL, Meng XW, Schneider PA, Knorr KLB (2018). Measurement of BH3-only protein tolerance. Cell Death Differ.

[46] Bentele M, Lavrik I, Ulrich M, Stosser S, Heermann DW, Kalthoff H (2004). Mathematical modeling reveals threshold mechanism in CD95-induced apoptosis. J Cell Biol.

[47] Kracikova M, Akiri G, George A, Sachidanandam R, Aaronson SA (2013). A threshold mechanism mediates p53 cell fate decision between growth arrest and apoptosis. Cell Death Differ.

[48] Hirsh D, Oppenheim D, Klass M (1976). Development of the reproductive system of Caenorhabditis elegans. Dev Biol.

[49] Agarwal V, Bell GW, Nam JW, Bartel DP. Predicting effective microRNA target sites in mammalian mRNAs. *eLife* 2015;4:1–38. 10.7554/eLife.05005 PMC4532895 26267216

[50] Hammell M, Long D, Zhang L, Lee A, Carmack CS, Han M (2008). mirWIP: microRNA target prediction based on microRNA-containing ribonucleoprotein-enriched transcripts. Nat Methods.

[51] D’Agostino I, Merritt C, Chen PL, Seydoux G, Subramaniam K (2006). Translational repression restricts expression of the C. elegans Nanos homolog NOS-2 to the embryonic germline. Dev Biol.

[52] Schmiedel JM, Klemm SL, Zheng Y, Sahay A, Bluthgen N, Marks DS (2015). Gene expression. MicroRNA control of protein expression noise. Science.

[53] Desvignes T, Pontarotti P, Fauvel C, Bobe J (2009). Nme protein family evolutionary history, a vertebrate perspective. BMC Evol Biol.

[54] Steeg PS, Bevilacqua G, Kopper L, Thorgeirsson UP, Talmadge JE, Liotta LA (1988). Evidence for a novel gene associated with low tumor metastatic potential. J Natl Cancer Inst.

[55] McCorkle JR, Leonard MK, Kraner SD, Blalock EM, Ma D, Zimmer SG (2014). The metastasis suppressor NME1 regulates expression of genes linked to metastasis and patient outcome in melanoma and breast carcinoma. Cancer Genomics Proteomics.

[56] Liu YF, Yang A, Liu W, Wang C, Wang M, Zhang L (2015). NME2 reduces proliferation, migration and invasion of gastric cancer cells to limit metastasis. PLoS ONE.

[57] Biggs J, Hersperger E, Steeg PS, Liotta LA, Shearn A (1990). A Drosophila gene that is homologous to a mammalian gene associated with tumor metastasis codes for a nucleoside diphosphate kinase. Cell.

[58] Dammai V, Adryan B, Lavenburg KR, Hsu T (2003). Drosophila awd, the homolog of human nm23, regulates FGF receptor levels and functions synergistically with shi/dynamin during tracheal development. Genes Dev.

[59] Hartsough MT, Morrison DK, Salerno M, Palmieri D, Ouatas T, Mair M (2002). Nm23-H1 metastasis suppressor phosphorylation of kinase suppressor of Ras via a histidine protein kinase pathway. J Biol Chem.

[60] Salerno M, Palmieri D, Bouadis A, Halverson D, Steeg PS (2005). Nm23-H1 metastasis suppressor expression level influences the binding properties, stability, and function of the kinase suppressor of Ras1 (KSR1) Erk scaffold in breast carcinoma cells. Mol Cell Biol.

[61] Lee MY, Jeong WJ, Oh JW, Choi KY (2009). NM23H2 inhibits EGF- and Ras-induced proliferation of NIH3T3 cells by blocking the ERK pathway. Cancer Lett.

[62] Tso PH, Wang Y, Yung LY, Tong Y, Lee MM, Wong YH (2013). RGS19 inhibits Ras signaling through Nm23H1/2-mediated phosphorylation of the kinase suppressor of Ras. Cell Signal.

[63] Fricker M, O’Prey J, Tolkovsky AM, Ryan KM (2010). Phosphorylation of Puma modulates its apoptotic function by regulating protein stability. Cell Death Dis.

[64] Datta SR, Dudek H, Tao X, Masters S, Fu H, Gotoh Y (1997). Akt phosphorylation of BAD couples survival signals to the cell-intrinsic death machinery. Cell.

[65] Filipowicz W, Bhattacharyya SN, Sonenberg N (2008). Mechanisms of post-transcriptional regulation by microRNAs: are the answers in sight?. Nat Rev Genet.

[66] Hu W, Coller J (2012). What comes first: translational repression or mRNA degradation? The deepening mystery of microRNA function. Cell Res.

[67] Gallo CM, Munro E, Rasoloson D, Merritt C, Seydoux G (2008). Processing bodies and germ granules are distinct RNA granules that interact in C. elegans embryos. Dev Biol.

[68] Wang JT, Seydoux G (2014). P granules. Curr Biol.

[69] Rajyaguru P, Parker R (2009). CGH-1 and the control of maternal mRNAs. Trends Cell Biol.

[70] Navarro RE, Shim EY, Kohara Y, Singson A, Blackwell TK (2001). cgh-1, a conserved predicted RNA helicase required for gametogenesis and protection from physiological germline apoptosis in C. elegans. Development.

[71] Brenner S (1974). The genetics of Caenorhabditis elegans. Genetics.

[72] Dickinson DJ, Pani AM, Heppert JK, Higgins CD, Goldstein B (2015). Streamlined genome engineering with a self-excising drug selection cassette. Genetics.

[73] Lant B, Derry WB (2014). Visualizing apoptosis in embryos and the germline of Caenorhabditis elegans. Cold Spring Harbor protocols.

[74] Chen C, Ridzon DA, Broomer AJ, Zhou Z, Lee DH, Nguyen JT (2005). Real-time quantification of microRNAs by stem-loop RT-PCR. Nucleic Acids Res.

[75] Das PP, Bagijn MP, Goldstein LD, Woolford JR, Lehrbach NJ, Sapetschnig A (2008). Piwi and piRNAs act upstream of an endogenous siRNA pathway to suppress Tc3 transposon mobility in the Caenorhabditis elegans germline. Mol Cell.

