# Supplementary material for: MiR-35 buffers apoptosis thresholds in the C. elegans germline by antagonizing both MAPK and core apoptosis pathways
Source: Cell Death Differ. 2019 Apr 5;26(12):2637–51. doi: 10.1038/s41418-019-0325-6 (PMC7224216; doi:10.1038/s41418-019-0325-6)
Supplement: Supplementary file 7 — Supplementary Table 1 [file 41418_2019_325_MOESM7_ESM.docx]

Supplementary Table 1: Primers used in the study. Sequences are written as 5’ 🡪 3’.

| LNA-probe: | |
| --- | --- |
| miR-35 | BioTEG/ACTGC+TAGTT+TCC+ACCCGGTGA |
| Small RNA real time PCR: | |
| miR-35 RT | CTCAACTGGTGTCGTGGAGTCGGCAATTCAGTTGAGACTGCTAG |
| miR-36 RT | CTCAACTGGTGTCGTGGAGTCGGCAATTCAGTTGAGCATGCGAA |
| miR-37 RT | CTCAACTGGTGTCGTGGAGTCGGCAATTCAGTTGAGACTGCAAG |
| miR-38 RT | CTCAACTGGTGTCGTGGAGTCGGCAATTCAGTTGAGACTCCAGT |
| miR-39 RT | CTCAACTGGTGTCGTGGAGTCGGCAATTCAGTTGAGCAAGCTGA |
| miR-40 RT | CTCAACTGGTGTCGTGGAGTCGGCAATTCAGTTGAGTTAGCTGA |
| miR-41 RT | CTCAACTGGTGTCGTGGAGTCGGCAATTCAGTTGAGTAGGTGAT |
| miR-42 RT | CTCAACTGGTGTCGTGGAGTCGGCAATTCAGTTGAGTCTGTAGA |
| sn2841 RT (internal control) | CTCAACTGGTGTCGTGGAGTCGGCAATTCAGTTGAGCTGCTCAG |
| miR-35 F | ACACTCCAGCTGGGTCACCGGGTGGAAACT |
| miR-36 F | ACACTCCAGCTGGGTCACCGGGTGAAAATT |
| miR-37 F | ACACTCCAGCTGGGTCACCGGGTGAACACT |
| miR-38 F | ACACTCCAGCTGGGTCACCGGGTGAACACT |
| miR-39 F | ACACTCCAGCTGGGTCACCGGGTGTAAATC |
| miR-40 F | ACACTCCAGCTGGGTCACCGGGTGAACACT |
| miR-41 F | ACACTCCAGCTGGGTCACCGGGTGAACACT |
| miR-42 F | ACACTCCAGCTGGGTCACCGGGTGAACACT |
| sn2841 F (internal control) | ACACTCCAGCTGGGCTGCGGTGACGATCAA |
| CRISPR: | |
| Ndk-1 sgRNA-n | TGTTGACACCTGTTCCAAGTGTTTTAGAGCTAGAAATAGCAAGT |
| Ndk-1-N 5’arm F | acgttgtaaaacgacggccagtcgccggcataaacatagggcgtcgaagg |
| Ndk-1-N 5’arm R | TCCAGTGAACAATTCTTCTCCTTTACTCAT CAT TTT CGG ACA ATC AAA CTT GG |
| Ndk-1-N 3’armF | CGTGATTACAAGGATGACGATGACAAGAGAAGCAACACTGAGAGAACCTTCA |
| Ndk-1-N 3’armR: | tcacacaggaaacagctatgaccatgttatTTC GTA GAC CCA TGA GTT GAT G |
| Site-directed mutagenesis of mir-35 binding site | |
| mir-35seedQ5F | tcttcatttgtgattatttttcgcagtcTCCGTCTCCAACTCCCCT |
| mir-35seedQ5R | GAAATCATGGTACAAATTGGAG |
