# Supplementary material for: MiR-35 buffers apoptosis thresholds in the C. elegans germline by antagonizing both MAPK and core apoptosis pathways
Source: Cell Death Differ. 2019 Apr 5;26(12):2637–51. doi: 10.1038/s41418-019-0325-6 (PMC7224216; doi:10.1038/s41418-019-0325-6)
Supplement: Supplementary file 8 — Supplementary Figure Legends [file 41418_2019_325_MOESM8_ESM.docx]

## Supplementary Figures

**Supplementary Figure 1. Inhibition of miR-35 activity leads to increase in germ cell death.** Cell death result in distinct morphological changes that can be observed under DIC microscopy. Young adult worms of N2, *mir-35(gk262)*, *ndk-1*::GFP, and *ndk-1*::GFP (*mir-35* mut) were irradiated at 0 Gy or 60 Gy and cell corpses imaged at 60X oil objective. White asterisks indicate these germ cell corpse bodies. Scale bar = 100 μm

**Supplementary Figure 2. Greater p-MPK-1 expression in the pachytene region of *mir-35(gk262)* relative to wild-type N2.** Young adult *mir-35(gk262)* or N2 worms were irradiated at 0 Gy or 60 Gy. Germlines were dissected 2 hrs after irradiation and stained for p-MPK-1 expression. Scale bar is 100 μm.

**Supplementary Figure 3. Aberrant NDK-1 activity led to heightened p-MPK-1 expression in the pachytene region of *mir-35(gk262)* mutant.** L4 staged N2 and *mr-35(gk262)* worms were fed with the HT115 control or *ndk-1* RNAi for 24 h, and then irradiated as young adults at 0 Gy or 60 Gy. Germlines were dissected 2 hrs after irradiation and stained for p-MPK-1 expression. Scale bar is 100 μm.

**Supplementary Figure 4. Germ cell corpses have increased NDK-1::GFP expression.** Young adult *ndk-1*::GFP worms were irradiated at 60 Gy and cell corpses imaged at 60X oil objective. White arrows indicate these germ cell corpse bodies. Scale bar = 100 μm

**Supplementary Figure 5. Mir-35, -36, -40, -41, and -42 miRNA family members were upregulated after irradiation.** Young adult N2 worms were irradiated at 0 Gy or 60 Gy. Total RNA was isolated from whole worms and real time PCR were carried out with miRNA-specific primers to each of the *mir-35* miRNA family members. SEM error bars represent 2 biological replicates with 3 technical replicates each. P < 0.0001 (****), P = 0.0008 (***), P < 0.0453 (*)

**Supplementary Figure 6. Germline development and germ cell maturation was not affected in *mir-35* mutants.** Germlines were isolated from young adult worms of N2, *mir-35(gk262)*, *ndk-1*::GFP, and *ndk-1*::GFP (*mir-35* mut) and stained with DAPI to determine different stages of germ cell development in the germline. Samples were imaged at 60X oil objective. Scale bar = 100 μm.

**Supplementary Movies 1 and 2. Intense NDK-1::GFP expression precedes cell death in irradiated germ cells in *ndk-1*::GFP (*mir-35* mut)**. Two examples of increase NDK-1::GFP expression in two different worms. Young adult worms were irradiated at 60 Gy, immobilized on 2% agarose and 1 mM tetramisole, and continuously imaged for 2 hrs under 40X water objective. Images were taken at 6 images per minute with 1 μm Z-stack slices and whole worm image was stitched together.

**Supplementary Movie 3. Greater *egl-1* mRNA expression in *mir-35*(*gk262*) mutant after irradiation compared to N2.** Movies showing *egl-1* mRNA FISH probe through different focal planes of isolated germlines from (A) N2 and (B) *mir-35*(*gk262*) at 0 Gy and (C) N2 and (D) *mir-35*(*gk262*) at 60 Gy. Germlines were isolated at 2 h post-irradiation for *egl-1* mRNA. Confocal images were taken at 60X oil, Z-stack at 1 μm slices and germline images were stitched together.
